# Postabortion Family Planning Progress: The Role of Donors and Health Professional Associations

**DOI:** 10.9745/GHSP-D-18-00334

**Published:** 2019-08-22

**Authors:** Carolyn Curtis, Anibal Faundes, Ann Yates, Ingela Wiklund, Martha Bokosi, Maryjane Lacoste

**Affiliations:** a Bureau for Global Health, U.S. Agency for International Development, Ethiopia Mission, Addis Ababa, Ethiopia.; b International Federation of Gynecology and Obstetrics, London, UK.; c State University of Campinas, Sao Paulo, Brazil.; d International Confederation of Midwives, The Hague, Netherlands.; e Family Planning Team, Bill & Melinda Gates Foundation, Seattle, WA, USA.

## Abstract

Global leadership from donors and international professional associations has enabled postabortion family planning services to be scaled up worldwide through preservice education, clinical service delivery, and global health programming.

## BACKGROUND

Globally, an estimated 1 in 4 pregnancies ends in induced abortion,^1^ and nearly 20% of postabortion clients have had a previous abortion.^2^ In a large study conducted in 14 countries, more than half of postabortion clients expressed interest in using contraception, but only 1 in 4 left the facility with a contraceptive.^2^

Unsafe abortion caused 13% of maternal deaths in 1990, with a case fatality rate of 340 per 100,000 women receiving unsafe abortion.^3^ In response, postabortion care (PAC) was introduced at the United Nations International Conference on Population and Development, held in September 1994 in Cairo. World leaders, high-ranking officials, representatives of NGOs, and United Nations agencies agreed upon an action plan,^4^ which included the following goals:

- All governments and relevant intergovernmental and nongovernmental organizations should strengthen their commitment to women's health, to deal with the health aspect of unsafe abortion as a major public health concern, and to reduce the recourse to abortion through expanded and improved family planning services.
- Women who have unintended pregnancies should have ready access to reliable information and compassionate counseling.
- In all cases, women should have access to quality services for management of complications arising from abortion.
- Postabortion counseling, education, and voluntary family planning services should be offered promptly to protect women's health and to help to avoid repeat abortions.

Since 1990, donors, international professional associations, and development partners have worked collaboratively to reduce maternal mortality due to complications of miscarriage and unsafe abortion, by delivering PAC services. Major partners include government donors (e.g., UK Department for International Development, Swedish International Development Cooperation Agency, German Technical Cooperation), multilateral organizations (United Nations Population Fund, United Nations Children's Fund), and private foundations (The David and Lucile Packard Foundation, The Rockefeller Foundation, Erik E. and Edith H. Bergstrom Foundation), among others.^5^

Since 1990, many entities have worked collaboratively to reduce maternal mortality by delivering PAC.

This commentary focuses on the actions taken by the U.S. Agency for International Development (USAID) from 1994 to the present and those of the International Confederation of Midwives (ICM), the International Federation of Gynecology and Obstetrics (FIGO), and the Bill & Melinda Gates Foundation (the Gates Foundation) to address the reduction of unsafe abortion by deliberative attention to postabortion family planning.

## USAID ACTIONS

In 1994, USAID began funding PAC programs as a key intervention in preventing maternal deaths and reducing unplanned pregnancies that may result in repeat abortion. These programs support treatment of complications from miscarriage and incomplete abortion, provide voluntary family planning counseling and services, and engage the community to reduce future unintended pregnancies and repeat abortions (Table).

### Leadership

USAID's global leadership has supported PAC programs in more than 40 countries worldwide by galvanizing support from the World Health Organization (WHO), FIGO, ICM, the International Council of Nurses (ICN), and donor organizations. USAID shared evidence with Senior Program Officers of the Gates Foundation and Family Planning 2020 (FP2020) that by providing voluntary postabortion family planning counseling and services at the same time and location as treatment, the average postabortion contraceptive uptake across 15 countries increased from 32% to 69%.^6^ USAID's advocacy led the Gates Foundation to adopt postabortion family planning as one of its initiatives for reaching FP2020 goals—specifically, enabling 120 million more women and girls to voluntarily use contraceptives. The recognition of the importance and role of postabortion family planning by the Gates Foundation and its inclusion in the foundation's programming will assist in institutionalizing PAC programming, leading to its sustainability in countries worldwide.

**TABLE.** Postabortion Care Models, 1990 to Present

Initial PAC Model (1990)	Revised USAID PAC Model (2004)	Revised PAC Consortium^a^ Model (2004)
Element 1: Emergency treatment	Component 1: Emergency treatment	Element 1: Community and service partnerships
Element 2: Family planning counseling and services	Component 2: Immediate family planning counseling and services; where human and financial resources exist, referral and/or treatment of STI and HIV testing	Element 2: Counseling
Element 3: Linkage to other reproductive health services	Component 3: Community empowerment through community awareness and mobilization	Element 3: Treatment
		Element 4: Contraception and family planning methods
		Element 5: Reproductive and other health services

Abbreviations: PAC, postabortion care; STI, sexually transmitted infection; USAID, U.S. Agency for International Development.

a The PAC Consortium was established in 1993 by Ipas, Association for Voluntary Surgical Contraception (now EngenderHealth), Jhpiego, Pathfinder International, and International Planned Parenthood Federation to raise awareness within the reproductive health community about the need to address complications of unsafe abortion and miscarriage. Its founding vision was to promote PAC as an effective strategy for improving maternal health and also to encourage USAID, the United Nations Population Fund, and other international donors and agencies in the reproductive health and population field to address the issue of unsafe abortion in their policies and programs.

### Terminology

Over the years, as new actors began implementing PAC programs, various terms have been used to describe these programs. Several countries use the term “comprehensive postabortion care” to define PAC programs that include voluntary family planning counseling and services. The Gates Foundation uses the terminology “post-pregnancy family planning” as the platform for implementing postabortion and postpartum family planning programs. ICM uses the terminology “abortion-related care” in its *Essential Core Competencies for Basic Midwifery Education*, which includes skills for postabortion family planning counseling and services.^7^

### Statutes and Policy

USAID-supported PAC programs include emergency treatment for complications of induced or spontaneous abortion, counseling on and provision of family planning options, and community mobilization. These programs do not promote abortion as a method of family planning. USAID-funded PAC programs are guided by multiple statutory and policy requirements that include restrictions related to abortion. Additionally, the principles of voluntarism and informed choice articulated in legislative and policy requirements guide USAID's family planning program, including PAC. PAC is explicitly permitted in the standard provision that implements the Protecting Life in Global Health Assistance policy (formerly the Mexico City Policy).^8^^–^^10^

### PAC Working Group

**FIGURE 1:**
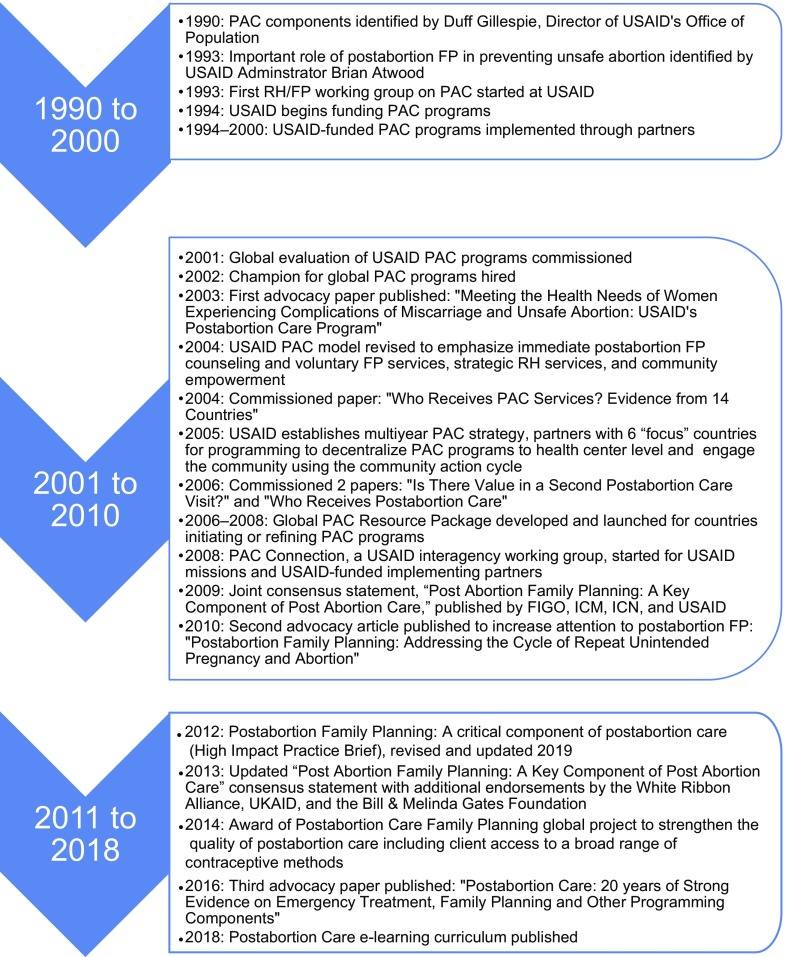
Key USAID Postabortion Care Activities, 1990 to 2018 Abbreviations: FIGO, International Federation of Gynecology and Obstetrics; FP, family planning; ICM, International Federation of Midwives; ICN, International Council of Nurses; PAC, postabortion care; RH, reproductive health; USAID, U.S. Agency for International Development.

In 1993, at a meeting of USAID cooperating agencies, USAID Administrator Brian Atwood highlighted the important role of family planning in preventing unsafe abortion and spoke of the need to work for “compassionate treatment of all women who are in such desperate circumstances that they are driven to seek an unsafe abortion” (written communication, Brian Atwood, 1993; Figure 1). In March 1993, the first reproductive health/family planning working group on PAC was convened by USAID. In 1994, USAID authorized the use of population funds for PAC and treatment for the first time, with family planning as the top priority.^11^

In 1993, USAID Administrator Brian Atwood highlighted the important role of family planning in preventing unsafe abortion.

### PAC Program Evaluation

In 2001, USAID/Washington commissioned a global evaluation of its PAC program with the purpose of conducting “a comprehensive and thorough review and analysis of the outstanding programmatic and technical issues in the current PAC portfolio.”^5^ At the time of the evaluation, PAC was being provided by USAID and others in 40 countries. Findings revealed that the greatest success was in the first component, treatment of abortion complications. The second component, voluntary family planning counseling and services, was not implemented as well as emergency treatment, and needed to be strengthened. The case studies and literature highlighted several continuing challenges, including the need to change attitudes among both providers and clients, and management and organizational issues, such as the availability of family planning commodities. A recommendation for “comprehensive postabortion care” was made in an effort to ensure the delivery of voluntary family planning services for women. Another recommendation was that PAC programs should devote more attention to training, monitoring, and supervision of health care providers with regard to family planning knowledge, supportive attitudes, and technical skills to ensure high-quality family planning provision; organization of family planning services (physical location and space); counseling; information, education, and communication materials; contraceptive supply and method choice; privacy; and integration with providers of emergency care.^5^

### Global PAC Strategy

**FIGURE 2:**
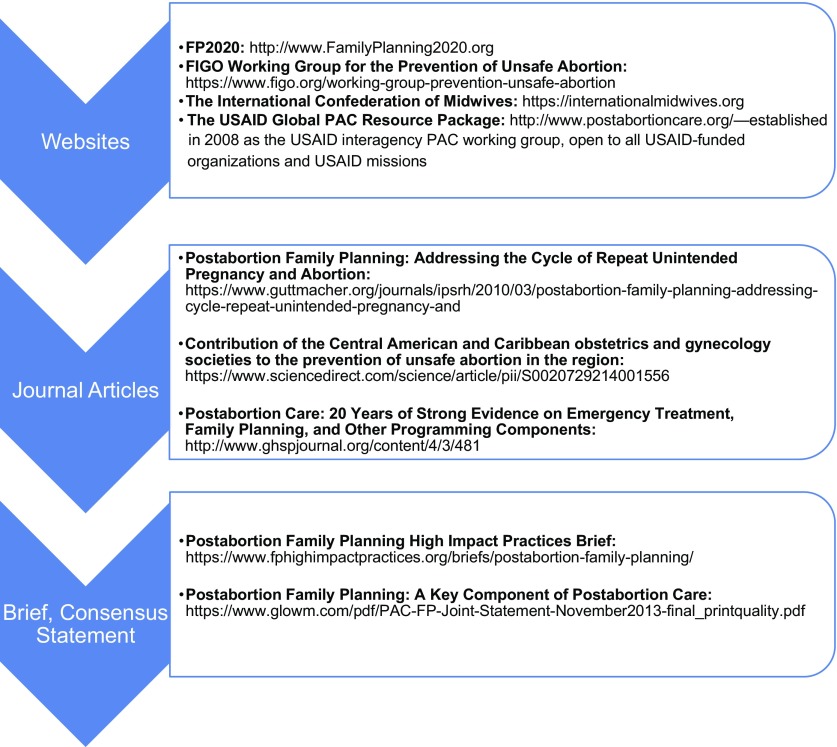
Key Resources for Postabortion Family Planning Abbreviations: FIGO, International Federation of Gynecology and Obstetrics; FP2020, Family Planning 2020; PAC, postabortion care.

In response to the 2001 global evaluation, the USAID PAC working group developed a multiyear strategy during 2003 and 2004 to advance and support the increased use of PAC with a particular focus on family planning counseling and services. Deliberative action was taken to address the challenges found in the global evaluation. Activities under the new strategy included (Figure 1 and Figure 2):

- Revision of the PAC model and results framework and indicators
- Selection of 6 focus countries for intensive support for PAC programming
- Establishment of the PAC Advisory Panel for the USAID PAC Working Group
- Development of a global PAC package, the “Global Resource Package”
- Establishment of the PAC Connection, a technical working group for USAID-funded implementing partners and USAID headquarters and field staff
- Publication of “Post Abortion Family Planning: A Key Component of Post Abortion Care” consensus statement^12^; a Family Planning High Impact Practice (HIP) Brief on Postabortion Family Planning^6^; and peer-reviewed articles in global journals

The USAID PAC working group developed a multiyear strategy to advance and support the increased use of PAC.

These activities addressed key themes within the PAC strategy, including the following:

- Standardization of training materials, guidelines, and indicators
- Expansion and institutionalization of PAC at the country level
- Identification of successful models by working intensely in focus countries
- Leadership to identify further research needs
- Compilation of research findings regarding the impact of PAC programs
- Dissemination of this information to donors
- Monitoring and evaluation

Key relationships were developed during the strategy period with international professional associations, such as FIGO, ICM, and ICN, to standardize global PAC policies, expand the reach of the PAC model, and promote the best practices in implementing PAC programs. Three models that evolved from work in the focus countries included (1) the decentralization of PAC from hospitals to health centers, (2) increased community empowerment through use of the community action cycle, and (3) validation that the provision of family planning services immediately after treatment and before discharge from the facility increased uptake of voluntary postabortion family planning. These models were piloted and replicated in a number of countries. Other key activities accomplished during the strategy period are outlined in Figure 1.

Key relationships were developed to standardize global PAC policies, expand their reach, and promote best practices.

## FIGO ACTIONS

Since January 2007, FIGO promoted family planning by establishing (1) the Committee on Contraception; (2) the Post-Partum IUD Program; and (3) the FIGO Working Group on the Prevention of Unsafe Abortion to promote family planning. Under the working group, FIGO started the Initiative on the Prevention of Unsafe Abortion (Box 1), which was aimed at reducing maternal mortality and morbidity associated with unsafe abortion and its burden for women.

BOX 1 **Goals of the International Federation of Gynecology and Obstetrics Working Group on the Prevention of Unsafe Abortion**

- Build national and international consensus to overcome constraints to providing modern contraception, to reduce the burden of unsafe abortion
- Increase awareness of obstetrics/gynecology professionals about their ethical obligations to increase women's access to contraceptive methods
- Promote and advance women's access to postabortion services
- Develop and issue statements, position papers, guidelines, and policy documents on various topics with other global organizations

In September 2009, FIGO together with the ICM, ICN, and USAID signed the first joint consensus statement entitled “Family Planning: A Key Component of Post Abortion Care.”^13^ FIGO used this statement to advocate for the inclusion of voluntary postabortion family planning services when treating abortion complications.

Using the joint consensus statement, a global project was started at the country level under the Initiative to Prevent Unsafe Abortion with FIGO's member societies to address maternal mortality due to unsafe abortion. FIGO's central headquarters provided financial and technical assistance. The project consisted of 2 phases, which had country and regional activities.

### Phase I, Step 1: Country Activities

1. FIGO member societies in countries with an induced abortion rate of 30 per 1,000 among women ages 15–44 or an unsafe abortion rate of 10 per 1,000 were invited to participate in the initiative.
2. Participating countries in the project identified a focal point and conducted a situational analysis of unsafe abortion in their country.
3. After completion of the analysis, each country held a national workshop with the participation of the government and interested parties. The results were discussed and a country action plan was developed to respond to the deficiencies. The action plan was adopted as a country commitment by the government and civil society.

### Phase I, Step 2: Regional Activities

FIGO's headquarters organized regional workshops for countries in their respective regions. Each participating country presented the various problems encountered in their country related to unsafe abortion and the actions taken to solve them. Country action plans were refined at the regional workshop, and government representatives attending the regional workshops were asked to commit themselves to implementing the action plans during the ensuing 2 years.

### Phase II

Country action plans developed at the regional workshops were implemented. Phase II began with the launching of the action plan and at a minimum will end when the goal of reducing unsafe abortion is reached.

### Implementation

When the global project began in 2008, 40 countries accepted the invitation to participate. Initially, only 26 of 40 member societies included voluntary postabortion contraception in their action plans. From 2008 to 2016, regional workshops were held annually in which the progress against the country action plans was reviewed with representatives of the member societies and the respective Ministries of Health.

The regional workshops showed 2 ways in which postabortion contraception activities could be more effective: (1) offering a choice of contraceptive methods before the patient's discharge, and (2) including the option of voluntary long-acting reversible contraceptives (LARCs). One presentation entitled “The role of post abortion contraception to reduce unsafe abortion” was prepared to emphasize the importance of voluntary postabortion contraception to reduce unsafe abortion.^14^ By 2013, all 40 countries included voluntary postabortion contraception in their action plans.

By 2013, all 40 FIGO member countries included voluntary postabortion contraception in their action plans.

Although advocacy for postabortion contraception was important, the member societies also learned that more could be achieved by introducing postabortion contraception as a new clinical service in hospitals and by introducing postabortion contraceptive services into teaching hospitals and in the training curriculum of residents. The ability of the national member societies to initiate or improve the provision of postabortion contraceptive services was reflected in the radical changes seen in their new action plans. The action plans included what each society or its prominent members could do through teaching hospitals to act directly on the provision of family planning services. In addition, several societies were active in providing family planning training for public health providers.

As of 2017, immediate postabortion contraceptive services are provided in teaching hospitals in 46 participating countries. In 8 Caribbean and Central American countries, updated guidelines are now in use, and emphasis is being placed on postabortion contraception counseling and the provision of voluntary contraceptive methods. Honduras and Nicaragua have included training on the provision of intrauterine devices and implants to increase their voluntary use by postabortion patients.^15^

## ICM ACTIONS

The global deficit of skilled health care professionals is estimated to reach 12.9 million by 2035.^16^ Such shortages are especially critical in regions of the world that also have a high burden of unsafe abortion and related maternal mortality. Globally, an acknowledged inequity exists in access to trained, skilled professional midwives. The vision of ICM focuses on ensuring that all women, irrespective of their economic status, have access to a midwife's care for herself and her newborn. This factor is key to reducing maternal, newborn, and infant mortality. ICM plays a major role in preparing midwives via preservice education to care for women who have experienced miscarriage or induced abortion. One of ICM's position statements, “Midwives’ provision of abortion-related services,”^17^ notes that midwives should “provide the woman (and where appropriate her family) with education concerning her future health, including contraception and planning for future pregnancy.”

ICM's *Essential Competencies for Basic Midwifery Practice: 2018 Update*^7^ outlines 4 areas of competency and provides the basic postabortion family planning skills and/or abilities for midwives completing preservice education programs. Competency 2i, “Provide Care to Women with Unintended or Mistimed Pregnancy,” centers on the knowledge of family planning methods appropriate for the postabortion period and skills for postabortion family planning, including reviewing with women options for contraception and helping them to initiate immediate use of an appropriate method.

Currently, ICM is working with a USAID-supported partner to develop modules for PAC and family planning for competency-based global midwifery curricula. The modules will be used by midwifery faculty in preservice education. This activity includes the training of midwifery educators in competency-based methodologies using LARC and PAC modules. Newly qualified graduates in the near future are expected to have the knowledge, skills, and attitudes to provide a wide choice of contraceptive methods, including LARCs, as well as high-quality, safe PAC. ICM has also developed standards related to family planning and PAC for countries to use in developing their midwifery curricula. The competency-based modules for PAC and family planning are expected to be available to countries in 2020.

## THE GATES FOUNDATION ACTIONS

The family planning goals of the Gates Foundation align with the global commitments of the 2012 London Summit on Family Planning, which are to ensure access to high-quality contraceptive information, services, and supplies so that an additional 120 million women and girls in the poorest countries are voluntarily using modern contraception by 2020, a milestone on the pathway toward universal access. These goals were reaffirmed in 2017.

The family planning goals of the Gates Foundation align with the global commitments of the 2012 London Summit on Family Planning.

In November 2013, the Gates Foundation, along with USAID, UKAID, the White Ribbon Alliance, FIGO, ICM, and ICN, signed the revised joint consensus statement on the critical role of postabortion family planning as part of PAC, recognizing this area of work as a key contributor to FP2020, the Millennium Development Goals, and the Sustainable Development Goals.^12^ To support the commitments of the joint consensus statement, all endorsing organizations agreed to work with FP2020 governments through their grantees and partners to reconfigure postabortion family planning services for maximum results, provide an expanded contraceptive method mix, emphasize and improve counseling to ensure voluntarism and informed choice, and support follow-up to optimize contraceptive continuation post procedure (Box 2). Postabortion family planning services can be universally offered within both safe abortion and PAC settings.

BOX 2 **Commitments Made in the Joint Consensus Statement on Postabortion Family Planning**

We commit ourselves and call upon all programs serving postabortion women of all ages to:

- Ensure that voluntary family planning counseling and services are included as an essential component of postabortion care in all settings
- Empower and serve postabortion women of all ages to prevent unintended pregnancies and further abortions
- Provide information on optimal pregnancy spacing for those women who want a pregnancy in order to realize critical health benefits, such as reduced maternal, neonatal, and childhood deaths, and prevention of HIV transmission from mother to child

We recognize that postabortion family planning is a cost-effective strategy for helping countries meet their commitments under Millennium Development Goal 5; FP2020; A Promise Renewed and the United Nations General Assembly Special Session on HIV/AIDS (UNGASS).

The International Federation of Gynecology and Obstetrics (FIGO), the International Confederation of Midwives (ICM), and the International Council of Nurses (ICN) have committed to fully collaborate across their professions to optimize the provision of postabortion family planning, and through this statement, they are joined by collaborating partners to achieve universal access to voluntary postabortion family planning.

Published in 2013 by the International Confederation of Midwives, the International Council of Nurses, the Unites States Agency for International Development, the White Ribbon Alliance, the Department for International Development, and the Bill & Melinda Gates Foundation.^12^

### Uniting Postabortion and Postpartum Family Planning Initiatives

In most low-resource settings, treatment for postabortion complications often occurs in the same location where deliveries are performed by skilled birth attendants. As countries strengthen their national programs to offer family planning to postpartum women, they should ensure that postabortion clients are counseled and offered voluntary family planning services. Including postabortion family planning when introducing postpartum family planning in health facilities makes sense both programmatically and technically. Thus, the Gates Foundation has committed to addressing postabortion and postpartum family planning through a common framework of post-pregnancy family planning.

As part of its priority programming for 2018–2020, the Gates Foundation continues to invest in postabortion family planning programs in its 9 priority countries. The Gates Foundation supports service delivery efforts at national and subnational levels, as well as innovative operations research on best practices for ensuring that women who seek medical abortion, both within and outside health facilities, have immediate access to a broad range of postabortion contraceptive counseling and voluntary services. Special interest exists for developing innovative solutions for women who access pharmacies or drug shops where immediate access to postabortion family planning could be expanded. Examples of current investments are noted in Box 3.

BOX 3 **Posabortion Activities of the Bill & Melinda Gates Foundation**

- **Democratic Republic of the Congo:** In partnership with other donors, the ExpandFP II project is providing technical assistance to the national Ministry of Health to integrate postpartum and postabortion family planning countrywide into maternal, newborn, and child health services through development of costed planning and implementation documents; establishment of structures and processes for collaborative planning, implementation, and coordination; documentation and dissemination of best practices; and promotion of limited pilots in other provinces.
- **Pakistan**: The Naya Qadam project is working to increase access to high-quality post-pregnancy family planning, and more specifically postabortion family planning, in both public and private sectors, with a focus on young women (ages 15–24) in Sindh and Punjab provinces. This program will operate at scale in 6 districts across Sindh and Punjab, reaching women with quality counseling and contraceptive method provision, and increasing method choice, including long-acting contraception.
- **India**: The Foundation is working with 7 states—Uttar Pradesh, Bihar, Assam, West Bengal, Maharashtra, Karnataka, and Haryana—to introduce and scale up comprehensive post-pregnancy services, leveraging the introduction of new methods (such as injectables) to ensure the availability of a contraceptive method mix that is as broad as possible. Foundation-funded partners are supporting these states to incorporate budgetary support into local program implementation plans to continue to take programs to scale at the subnational level.
- **Nigeria:** The Foundation is supporting a program in the states of Lagos, Nasarawa, and Rivers to enable access to family planning information and services to provide comprehensive postabortion care, including family planning, following spontaneous or induced abortions.
- **Operations research in Kenya and Indonesia**: In collaboration with Merck for Mothers, the Foundation is supporting an implementation research study to better understand post-pregnancy barriers and facilitators and to generate actionable evidence for realistic policy and programmatic efforts to address them in both the public and private sectors.

The global postpartum family planning steering committee, which was established in 2014 to support the organization of the 2015 Chiang Mai global postpartum family planning meeting, continues to be supported through the Gates Foundation. As part of the FP2020 secretariat's mandate, the postpartum family planning component has been expanded to also address postabortion family planning, with FP2020 now including the following functions as part of its work:

- Regular coordination calls for postpartum/postabortion family planning global steering committee (comprising all donors active in this space, as well as other critical stakeholders, such as WHO)
- Webinars and/or other knowledge platforms for countries to share best practices/experiences/issues/requests for assistance including the dissemination of the postabortion family planning HIP brief^6^
- The inclusion of postpartum/postabortion family planning in regional focal point workshops and linking relevant technical partners when family planning needs are identified
- Working with countries to ensure postpartum/postabortion family planning efforts are incorporated into and tracked against national family planning costed implementation plans, Global Financing Facility investment cases, and FP2020 action plans
- Coordination with postabortion family planning around data/program information use for postabortion family planning and postpartum family planning advocacy purposes

In addition, the Gates Foundation has provided funding to FP2020's Rapid Response Mechanism for dedicated support of country-level catalytic postpartum/postabortion family planning activities. The Foundation looks forward to its continued work with the global family planning community to ensure that there are no missed opportunities to give women increased access to their preferred contraceptive methods where and when they want them.

## CONCLUSION

The bold and compassionate actions of these organizations and professional associations with their governments have saved the lives of countless women and averted harm for countless more. Their efforts have produced the evidence-based practices for increasing the voluntary uptake of postabortion family planning as a lifesaving intervention to be mainstreamed, institutionalized, and sustained throughout the world. Continued collaboration and commitment across governments, health professional associations, and international organizations will be vital for achieving universal access to postabortion family planning as part of PAC. Together with postpartum family planning, postabortion family planning will make vital contributions to maternal health, the goals of FP2020, and the Sustainable Development Goals. Now is the time for renewed effort and strong collaboration.

Continued collaboration and commitment will be vital for achieving universal access to voluntary postabortion family planning.

## Notes

**Peer Reviewed**

**Funding:** None.

## References

[1] Preventing unsafe abortion. World Health Organization website https://www.who.int/news-room/fact-sheets/detail/preventing-unsafe-abortion. Published February 19, 2018 Accessed April 8, 2019.

[2] Kidder E Sonneveldt E Hardee K Who Receives PAC Services? Evidence from 14 Countries. Washington, DC: The Futures Group; 2004.

[3] World Health Organization (WHO). Unsafe abortion incidence and mortality: global and regional levels in 2008 and trends during 1990–2008. Geneva: WHO; 2012 https://apps.who.int/iris/handle/10665/75173. Accessed April 8, 2019.

[4] United Nations Population Fund (UNFPA). Programme of Action Adopted at the International Conference of Population and Development, Cairo, 5–13 September, 1994. New York: UNFPA; 2004 https://www.unfpa.org/sites/default/files/event-pdf/PoA_en.pdf. Accessed April 8, 2019.

[5] Cobb L Buono N Dunlop J Global evaluation of USAID's postabortion care program. Washington, DC: Population Technical Assistance Project; 2001 https://pdf.usaid.gov/pdf_docs/PNACN773.pdf. Accessed April 8, 2019.

[6] High Impact Practices in Family Planning (HIP). Postabortion family planning: a critical component of postabortion care. Washington, DC: U.S. Agency for International Development; 2019 https://www.fphighimpactpractices.org/briefs/postabortion-family-planning/. Accessed April 24, 2019.

[7] International Confederation of Midwives (ICM). Essential Competencies for Midwifery Practice: 2018 Update. [The Hague, Netherlands; ICM; 2018]. https://www.internationalmidwives.org/assets/files/general-files/2018/10/icm-competencies—english-document_final_oct-2018.pdf. Accessed April 24, 2019.

[8] Trump DJ Presidential memorandum regarding the Mexico City Policy. The White House website https://www.whitehouse.gov/presidential-actions/presidential-memorandum-regarding-mexico-city-policy/. Issued January 23, 2017 Accessed April 8, 2019.

[9] Protecting Life in Global Health Assistance fact sheet. U.S. State Department website https://www.state.gov/r/pa/prs/ps/2017/05/270866.htm. Published May 15, 2017 Accessed April 8, 2019.

[10] Executive Office of the President. Memorandum of January 23, 2017. Mexico City Policy: Memorandum for the Secretary of State, the Secretary of Health and Human Services, and the Administrator of USAID at https://www.whitehouse.gov/presidential-actions/presidential-memorandum-regarding-mexico-city-policy/. Accessed April 24, 2019.

[11] Curtis C. Meeting health care needs of women experiencing complications of miscarriage and unsafe abortion: USAID's postabortion care program. J Midwifery Womens Health. 2007;52(4):368–375. 10.1016/j.jmwh.2007.03.005. 17603959

[12] International Federation of Gynecology and Obstetrics (FIGO); International Confederation of Midwives (ICM); International Council of Nurses (ICN); U.S. Agency for International Development (USAID); White Ribbon Alliance; Department for International Development (DFID); Bill & Melinda Gates Foundation. Post abortion family planning: a key component of post abortion care. Consensus statement. https://www.figo.org/sites/default/files/uploads/project-publications/PAC-FP-Joint-Statement-November2013-final_printquality.pdf. Published November 1, 2013 Accessed April 24, 2019.

[13] International Federation of Gynecology and Obstetrics (FIGO); International Confederation of Midwives (ICM); International Council of Nurses (ICN); U.S. Agency for International Development (USAID). Family planning: a key component of post abortion care. Consensus statement. https://www.globalhealthlearning.org/sites/default/files/page-files/USAID_2009_fp_component.pdf. Published September 25, 2009 Accessed April 24, 2019.

[14] International Federation of Gynecology and Obstetrics (FIGO). The role of post abortion contraception in prevention of unsafe abortion [presentation]. https://www.figo.org/sites/default/files/uploads/OurWork/FIGO%20PUA%20WG%20-%20The%20role%20of%20post%20abortion%20contraception.pdf. Accessed April 8, 2019.

[15] de Gil MP Contribution of the Central American and Caribbean obstetrics and gynecology societies to the prevention of unsafe abortion in the region. Int J Gynaecol Obstet. 2014;126(suppl 1):S10–S12. 10.1016/j.ijgo.2014.03.005 24745695

[16] Global Health Workforce Alliance; World Health Organization (WHO). A Universal Truth: No Health Without A Workforce. Geneva: WHO; 2013 https://www.who.int/workforcealliance/knowledge/resources/hrhreport2013/en/. Accessed April 24, 2019.

[17] International Confederation of Midwives (ICM). Position statement: midwives’ provision of abortion-related services. The Hague, Netherlands: ICM; 2014 https://www.internationalmidwives.org/assets/files/statement-files/2018/04/midwives-provision-of-abortion-related-services-eng.pdf. Accessed April 8, 2019.

